# Usability and acceptability of oral-based HCV self-testing among key populations: a mixed-methods evaluation in Tbilisi, Georgia

**DOI:** 10.1186/s12879-022-07484-2

**Published:** 2022-05-31

**Authors:** Emmanuel Fajardo, Victoria Watson, Moses Kumwenda, Dali Usharidze, Sophiko Gogochashvili, David Kakhaberi, Ana Giguashvili, Cheryl C. Johnson, Muhammad S. Jamil, Russell Dacombe, Ketevan Stvilia, Philippa Easterbrook, Elena Ivanova Reipold

**Affiliations:** 1 The Foundation for Innovative New Diagnostics (FIND), Campus Biotech, Chemin des Mines 9, 1202 Geneva, Switzerland; 2 Liverpool School of Tropical Medicine (LSTM), Liverpool, UK; 3 Malawi Liverpool Wellcome Trust Clinical Research Programme (MLW), Blantyre, Malawi; 4 College of Medicine, University of Malawi (CoM), Blantyre, Malawi; 5 Non-Governmental Organization New Way, Tbilisi, Georgia; 6 Community-Based Organization Equality Movement, Tbilisi, Georgia; 7 National Centre for Disease Control and Public Health of Georgia, Tbilisi, Georgia; 8 Department of Global HIV, Hepatitis and STI Programmes, World Health Organization, Geneva, Switzerland

**Keywords:** HCV, Screening, Oral fluid, Self-testing, Acceptability, MSM, PWID, Georgia

## Abstract

**Background:**

Hepatitis C virus self-testing (HCVST) is an additional approach that may expand access to HCV testing. We conducted a mixed-methods cross-sectional observational study to assess the usability and acceptability of HCVST among people who inject drugs (PWID), men who have sex with men (MSM) and transgender (TG) people in Tbilisi, Georgia.

**Methods:**

The study was conducted from December 2019 to June 2020 among PWID at one harm reduction site and among MSM/TG at one community-based organization. We used a convergent parallel mixed-methods design. Usability was assessed by observing errors made and difficulties faced by participants. Acceptability was assessed using an interviewer-administered semi-structured questionnaire. A subset of participants participated in cognitive and in-depth interviews.

**Results:**

A total of 90 PWID, 84 MSM and 6 TG were observed performing HCVST. PWID were older (median age 35 vs 24) and had a lower level of education compared to MSM/TG (27% vs 59%). The proportion of participants who completed all steps successfully without assistance was 60% among PWID and 80% among MSM/TG. The most common error was in sample collection and this was observed more often among PWID than MSM/TG (21% vs 6%; p = 0.002). More PWID requested assistance during HCVST compared to MSM/TG (22% vs 8%; p = 0.011). Acceptability was high in both groups (98% vs 96%; p = 0.407). Inter-reader agreement was 97% among PWID and 99% among MSM/TG. Qualitative data from cognitive (n = 20) and in-depth interviews (n = 20) was consistent with the quantitative data confirming a high usability and acceptability.

**Conclusions:**

HCVST was highly acceptable among key populations in Georgia of relatively high educational level, and most participants performed HCVST correctly. A significant difference in usability was observed among PWID compared to MSM/TG, indicating that PWID may benefit from improved messaging and education as well as options to receive direct assistance when self-testing for HCV.

**Supplementary Information:**

The online version contains supplementary material available at 10.1186/s12879-022-07484-2.

## Background

Globally, in 2019 an estimated 58 million people were living with chronic hepatitis C virus (HCV) infection, and more than 300,000 people died from HCV-related liver disease [1]. Georgia is a lower-middle-income country in eastern Europe that has been highly affected by HCV. It has a high national HCV viraemic prevalence of 5.4% in the adult population, and with a population of around 3.7 million, there are an estimated 150,000 people living with HCV [2]. Most affected populations, include people who inject drugs (PWID) that account for more than one third of cases among the general population, and men who have sex with men (MSM). The prevalence of HCV antibody positivity among PWID is 65%–75%, and among MSM ranges between 7.1% and 18.9% in Georgia [3, 4]. HCV prevalence is higher among those HIV-coinfected [5, 6].

In response to their significant HCV public health problem, the government of Georgia launched in 2015 one of the world’s first nationwide HCV elimination programmes to decrease HCV viraemic prevalence by 90% by 2020 [7]. Ambitious targets were set to diagnose 90% of HCV-infected people, treat 95% of those diagnosed, and cure 95% of those treated with direct-acting antivirals (DAA) [8]. By December 2020, the national HCV screening programme tested around 2.2 million people, which is nearly 70% of the adult population (M Japaridze, personal communication, 2021). Despite this progress, there is still a substantial testing and treatment gap with 60,000 undiagnosed and untreated.

The 2017 World Health Organization (WHO) testing guidelines for hepatitis B and C recommend routine HCV testing for most affected populations that include PWID, MSM, persons in prison, as well as those settings and countries with a general population prevalence ≥ 2% [9, 10]. Although there are excellent examples of countries efforts in scaling up HCV testing services including facility and community-based testing [11, 12], bridging the diagnostic gap requires additional strategies to those still unaware of their HCV status or who have not yet accessed treatment. Future diagnostic innovations highlighted to promote access to HCV testing include the use of self-testing with oral or blood-based tests allowing individuals to test and interpret their own test results in a place of their choice, overcoming confidentiality and privacy issues [13]. HIV self-testing (HIVST) has been recommended by WHO since 2016 [14], and has enabled national HIV programmes to expand testing, especially among higher risk populations [15]. Accumulating evidence shows that HIVST is easy and safe [16], accurate [17], highly acceptable across different populations [18, 19], and increases testing uptake and linkage to care using different distribution strategies [20–22]. It has also been shown to be cost-effective in many settings; especially when focused on priority populations with lower testing and treatment coverage [23]. In July 2021, WHO released new guidelines strongly recommending offering HCV self-testing (HCVST) as an additional approach to HCV testing services [24]. HCVST can also be used to increase the frequency of testing among those testing negative, however, periodic retesting on people at ongoing risk and a history of treatment-induced or spontaneous clearance of HCV infection may be offered HCV RNA testing every 3–6 months, as the antibody remains positive after the first infection [9].

The OraQuick^®^ HCV Rapid Antibody Test Kit (OraSure, USA) is currently the only oral fluid-based test that has obtained WHO prequalification and market authorization in Europe and the United States [25, 26]. The accuracy of the professional OraQuick HCV test using oral fluids is high, with a reported pooled sensitivity of 98% (95% CI: 97–100) and specificity of 100% (95% CI: 100–100) [27]. A similar performance was observed in a recent study conducted in United States where the test was used for HCVST with test results being interpreted by trained staff: the sensitivity and specificity were 98% (95% CI: 88–100) and 98% (95% CI: 90–100), respectively [28]. However, when untrained users conducted the test and interpreted their own results, a lower sensitivity of 88% (95% CI: 75–96) was observed whilst specificity remained 100% (95% CI: 93–100) [28].

The literature on acceptability, values and preferences surrounding HCVST remains limited. However, some willingness to self-test was reported among PWID and MSM in England, with respondents noting potential benefits of HCVST included rapid results, ease to perform, and indicated preference for oral-based self-tests [29]. A study in Kyrgyzstan found that HCVST would be acceptable among PWID as a way to reduce stigma often encountered in healthcare facilities [30].

Further research is still needed on the usability and acceptability of HCVST in different populations and geographic regions to inform policy recommendations. To address this gap, FIND in collaboration with WHO has conducted a series of usability and acceptability studies among different populations in China, Egypt [31], Kenya, Malaysia, Rwanda, Pakistan and Vietnam [32]. In this study in Georgia, we sought to assess the usability and acceptability of oral-based HCVST among PWID and MSM/transgender people (TG).

## Methods

### Study design

We used a convergent parallel mixed-methods design [33] to assess the usability and acceptability of users in conduct of HCVST based on: correctly performing and interpreting test results, inter-reader and inter-operator agreement, acceptability and preferences for HCVST, current knowledge about HCV infection, testing and treatment services, and barriers and enablers to HCVST. The quantitative component entailed the direct observation of participants conducting HCVST and documentation of observed errors, difficulties and assistance required. The qualitative component was conducted using cognitive and in-depth interviews to complement quantitative data. All participants provided written informed consent. The local institutional review board approved the study protocol (#2019-066).

### Study settings and participant recruitment and eligibility

Two sites in Tbilisi participated in the study: a harm reduction site run by the non-governmental organization “New Way” and the lesbian, gay, bisexual, transgender and queer (LGBTQ +) community-based organization “Equality Movement”. PWIDs seeking services at “New Way” and MSM/TG at “Equality Movement” were approached to participate in the study. Assuming that 50% of eligible participants will accept HCVST, a minimum sample size of 100 participants in each study group was required to provide estimates with a 95% confidence interval and a margin error of ± 10%. Participants were eligible if they were 18 years or older, were able to read Georgian, did not know their HCV serostatus or tested negative > 1 year ago, provided written informed consent, and reported use of unprescribed intravenous drugs within the past year (PWID) or they had at least one anal sex episode with another man within the past year (MSM/TG). Individuals were excluded if they had had prior experience with HIVST. The first participants enrolled among PWID and MSM/TG were invited to participate in cognitive interviews (n = 20) and in-depth interviews (n = 20).

### Study procedures

The OraQuick^®^ HCV Rapid Antibody Test was used both for oral-based self-testing and professional oral-based testing. Although the test device is identical in both configurations, the external kit packaging and the instructions differ according to each intended use, as recommended by WHO [34]. For the OraQuick HCV Self-Test (Research-Use-Only), the packaging was simplified into a divided pouch containing also a disposal bag. The instructions were abbreviated into double-sided page containing pictorial instructions and simple nontechnical language (see Additional file 1). The instructions of the self-test was translated into Georgian.

The OraQuick^®^ HCV Rapid Test is a manually performed, visually read, 20-min immunoassay for the qualitative detection of HCV antibodies [26]. The oral-based self-testing workflow involves a 12-step process, namely: Pre-testing: (1) Opening the package, (2) Reading/using the instructions, (3) Removing the test tube from the test pack, (4) Removing the cap from the test tube, (5) Placing the tube into the stand, (6) Removing the test device from the test pack. Testing: (7) Correctly handling the device (not touching the flat pad), (8) Collecting oral fluid specimen, (9) Placing the test device in the test tube, (10) Checking test device stays in the tube while testing, (11) Time keeping for results. Post-testing: (12) Interpreting results and kit disposal.

### Quantitative methods

#### Data collection and analysis

Upon enrolment, a trained study staff member (healthcare worker: HCW) in each site administered a baseline questionnaire to participants to gather sociodemographic data (see Additional file 2). Participants were then given the HCV self-test kit and instructed to carry out the test and interpret the results on their own, relying solely on the instructions in Georgian. HCW observed participants perform the 12-step test process and completed a standardised product-specific checklist to document errors and difficulties observed during the procedure (see Additional file 3). Assistance was provided by the HCW only if the participant requested help after having exhausted all attempts to complete the testing step (usually, after 15 min). Once the self-test was completed and read by each participant, the HCW re-read the self-test and documented both results. This paired data served to calculate inter-reader agreement. Finally, the HCW performed and interpreted an additional professional use OraQuick^®^ HCV Rapid Test. This paired data, between the self-tester result and HCW result, were used to calculate inter-operator agreement. HCW also administered a post-testing questionnaire designed to capture information on participant’s experience during HCVST as well as acceptability and preferences (see Additional file 4).

Descriptive statistics were used to summarize the study population. Differences between study groups were compared with the X^2^-square test or Fischer’s exact test for categorical variables and the *t*-test for continuous variables. All statistical tests were two-sided with alpha 0.05. Usability was assessed by calculating the frequencies of mistakes, difficulties and assistance needed at each step of the testing procedure from opening the package to reading the results. Usability was defined as the percentage of participants who completed the testing steps without assistance and interpreted results correctly [35]. The percent agreement along with the Cohen’s Kappa coefficient was used to calculate inter-reader and inter-operator agreement, both including all test results and excluding paired invalid test results. Data was analysed with Stata/IC 16.1 (StataCorp, College Station, USA).

Acceptability was defined as the willingness of participants to use HCVST in the future and it was classified as high (≥ 67%), moderate (66–34%) or low (≤ 33%) [19]. Acceptability was further categorized as pre-acceptability or post-test acceptability if it was measured prior or after conducting self-testing [36]. Values and preferences were defined as participants’ views, concerns and preferences on HCVST use and delivery [19].

### Qualitative methods

#### Data collection and analysis of cognitive interviews

Two research assistants conducted the interviews in Georgian. Observations of participants conducting HCVST were conducted simultaneously with the cognitive interviews to visually assess the participant’s ability to correctly perform and interpret the HCVST. The cognitive interview thematic guide mirrored the steps depicted in the product-specific checklist (see Additional file 5). Interviews were audio recorded, translated into English, transcribed, and saved on password‐secured computers.

The data from the observations were tabulated and trends in test performance identified using a framework approach containing a matrix of problem areas. The analysis process of the cognitive interviews data entailed three researchers familiarizing themselves with the data, developing themes and codes based on the content of the manufacturers instructions, and presentation of a descriptive analysis of data.

#### Data collection and analysis of in-depth interviews

In-depth interviews were used to explore participants’ experiences and gain insights into their perceptions of the feasibility of HCVST to enable triangulation with data from the cognitive interviews as well as to explore acceptability. Participants were interviewed using a semi-structured interview topic guide (see Additional file 6) based on a guide previously developed for HIVST but modified for HCVST [37]. The topic guide consisted of open-ended questions seeking to capture different key issues around HCVST such as barriers and enablers, feasibility, improvement areas, and possible adverse events. The topic guide was translated into Georgian language and piloted before use in the study. Field notes and recorded interviews were directly translated and transcribed verbatim into English and saved on password‐secured computers.

A coding framework was developed inductively and coded using NVIVO 12 qualitative analysis software (see Additional file 7). Thematic data analysis methods were used to interrogate the data [38]. To investigate patterns across categories, similar units of categories were collapsed to generate broader themes around HCVST. Data were also triangulated across different scripts and different researchers who collected it to ensure validity. Quotes representing each theme were extracted from transcripts and reviewed again within the context of the full text of the interview. The results are presented as descriptive narratives highlighting important quotes.

## Results

### Characteristics of the study population

**Fig. 1:**
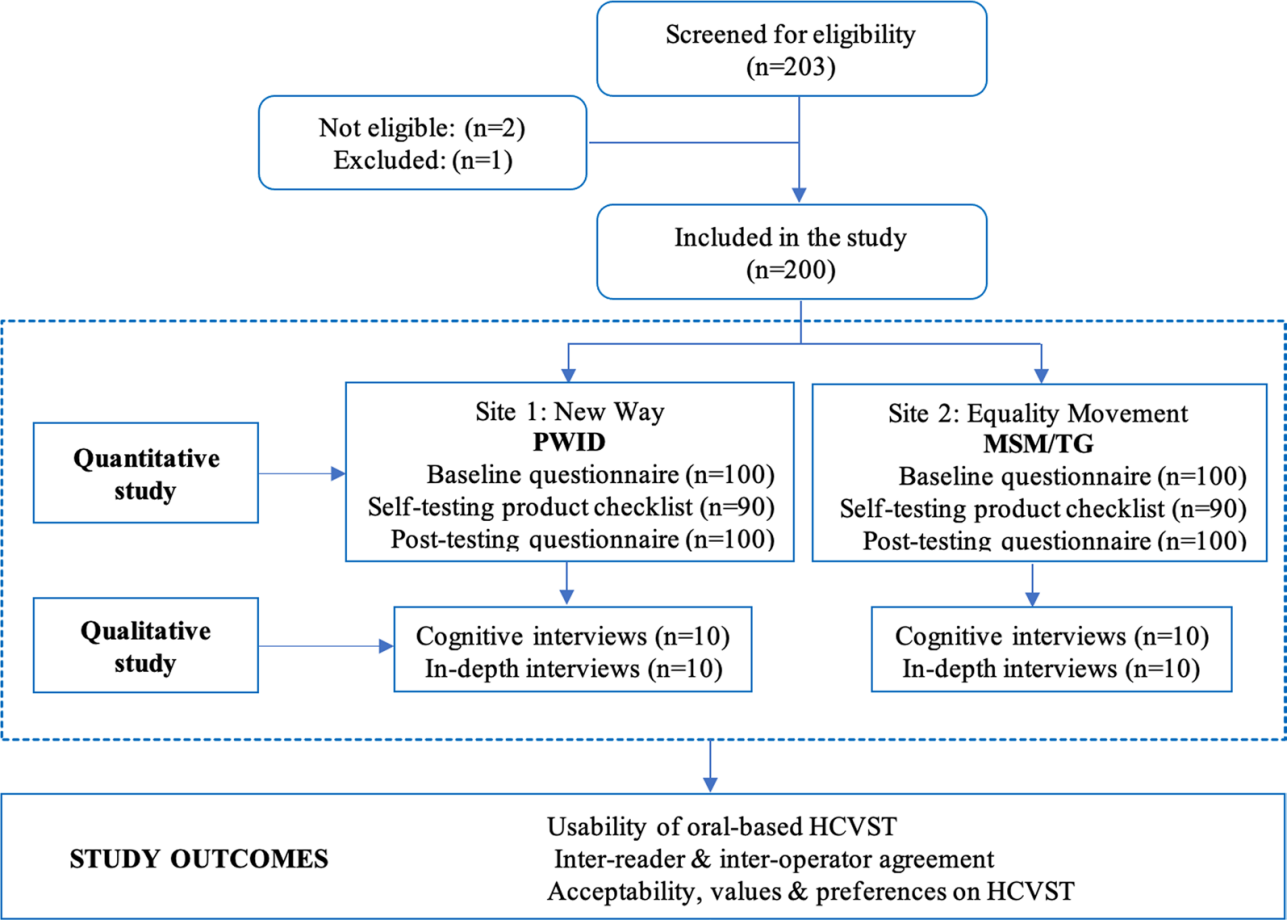
Flow diagram of individuals enrolled in the study

The number of participants enrolled in the study are shown in Fig. 1. A total of 100 PWID and 100 MSM/TG were enrolled in the study between December 2019 and June 2020 out of 203 individuals invited to participate (overall response rate was 98.5%). Baseline characteristics of the study populations are summarized in Table 1.

**Table 1:** Sociodemographic characteristics of study participants

Population group	PWID (n = 100)	MSM/TG (n = 100)*	*P* value^†^
Characteristics	n	n	
Median age, years (IQR)	35 (28–41)	24 (21–29)	< 0.001
Gender			
Male	98	91	0.004
Female	2	0	
Transgender	0	9	
Educational level			
Below high school	2	2	< 0.001
High school	58	19	
College	10	2	
Undergraduate	27	59	
Post graduate	3	18	
Employment			
Employed	41	74	< 0.001
Unemployed	59	25	
Missing data	0	1	
Marital status			
Married or living with a partner	28	5	< 0.001
Single	53	88	
Divorced or widowed	19	7	
Self-reported HCV risk factors			
Dental procedure(s)	75	58	0.011
Surgical procedure(s)	36	30	0.340
Sharing shaving tools/toothbrushes	12	17	0.315
Injecting unprescribed drugs	100	8	< 0.001
Sharing needles	33	1	< 0.001
Condomless anal sex	0	79	< 0.001
Has a tattoo	40	31	0.184
None reported	0	4	0.043
Frequency of routine health check			
More than once per year	3	57	< 0.001
Once per year	16	24	
Rarely	66	18	
Never	15	1	
Awareness about self-testing			
Aware certain tests can be done at home	32	96	< 0.001

*Nine participants self-identified as transgender people

^†^The two-sample t-test was used to compare median age and the Pearson’s chi-squared test for all categorical variables

PWID were older than MSM/TG (median age: 35 vs 24 year, p ≤ 0.001). The PWID population was predominantly male with only 2 women participating in the study. Nine of the MSM/TG individuals self-identified as transgender. There were some sociodemographic differences between MSM/TG and PWIDs that were statistically significant, namely: the MSM/TG population had a higher level of education (59.0% vs 27.0%, p ≤ 0.001), a higher proportion had a job (74.0% vs 41.0%, p ≤ 0.001), and most were aware of self-testing (96.0% vs 32.0%, p ≤ 0.001) compared to PWID. Eight participants among MSM/TG also reported injecting unprescribed drugs (8.0%).

For the sub-sample of 40 participants who underwent cognitive interview or in-depth interviews, the main sociodemographic characteristics between groups are presented in (see Additional file 8). Overall, MSM/TG were younger, more educated, tended to be employed and single compared to PWID. This subsample was representative of the entire sample because the sociodemographic characteristics in this subset of participants was consistent with that of the larger group reported in Table 1.

### Usability of the OraQuick HCV self-test

#### Quantitative findings

All participants were able to complete the entire HCVST procedure. The proportion of observed errors, difficulties and assistance provided in each study group is presented in Table 2. Among MSM/TG, 80% conducted all steps correctly and interpreted tests results accurately without assistance compared to 60% among PWID (p = 0.003). Difficulties were also more frequently observed among PWID than MSM/TG (34.4% vs 14.4%; p = 0.002). Few individuals interpreted the HCVST result incorrectly, 3.3% among PWID and 1.1% among MSM/TG (p = 0.062). Incorrect collection of the oral fluid specimen was the commonest error and this was observed more frequently among PWID than MSM/TG (21.1% vs 5.5%; p = 0.002), followed by inadequate time keeping to read the results (7.8% vs 0.0%; p = 0.014). Overall, assistance was requested and provided more often to PWID than MSM/TG (22.0% vs 7.8%; p = 0.011).

**Table 2:** OraQuick HCV Self-Test usability checklist: errors and difficulties

Population group	PWID (n = 90)		MSM/TG (n = 90)		*P* value
Observed errors	No	%	No	%	
*Pre-testing*					
1. Failed to open the package	0	0.0	0	0.0	–
2. Didn’t read/use the instructions	3	3.3	1	1.1	0.621
3. Didn’t remove the test tube from the test pack	0	0.0	0	0.0	–
4. Didn’t remove the cap from the test tube	0	0.0	1	1.1	0.316
5. Didn’t place the tube into the stand correctly	0	0.0	0	0.0	–
6. Didn’t remove the test device from the test pack	0	0.0	0	0.0	–
*Testing*					
7. Touched the flat pad	1	1.1	5	5.6	0.211
8. Incorrect procedure to collect oral fluid	19	21.1	5	5.6	0.002
9. Wrong placing of the test device in the test tube	0	0.0	1	1.1	0.316
10. Test device came out of the tube while testing	0	0.0	0	0.0	–
11. Inadequate time keeping	7	7.8	0	0.0	0.014
Errors observed for at least one step	25	27.8	15	16.7	0.073
*Post-testing*					
12. Interpreted the results incorrectly*	3	3.3	1	1.1	0.621
Observed difficulties					
1. Opening the package	0	0.0	6	6.7	0.013
4. Opening the tube	4	4.4	3	3.3	0.700
5. Placing the tube into the stand	3	3.3	1	1.1	0.621
8. Swabbing the mouth	4	4.4	3	3.3	0.700
9. Placing the device into the tube	4	4.4	1	1.1	0.174
12. Reading the test results	3	3.3	1	1.1	0.621
Experienced difficulties for at least one step	31	34.4	13	14.4	0.002
Assistance provided					
1. Opening the package	1	1.1	4	4.4	0.368
4. Opening and removing the cap from the tube	6	6.7	1	1.1	0.118
5. Placing the tube into the stand	5	5.6	2	2.2	0.444
9. Placing the test device into the tube	0	0.0	2	2.2	0.497
11. Reminding the incubation time	4	4.4	1	1.1	0.368
12. Reading the results	3	3.3	2	2.2	1.000
Assistance provided for at least one step	20	22.2	7	7.8	0.011
All steps completed correctly without assistance and test results reported correctly	54	60.0	72	80.0	0.003

*Disagreement between results of self-testers and trained staff re-reading

**Fig. 2:**
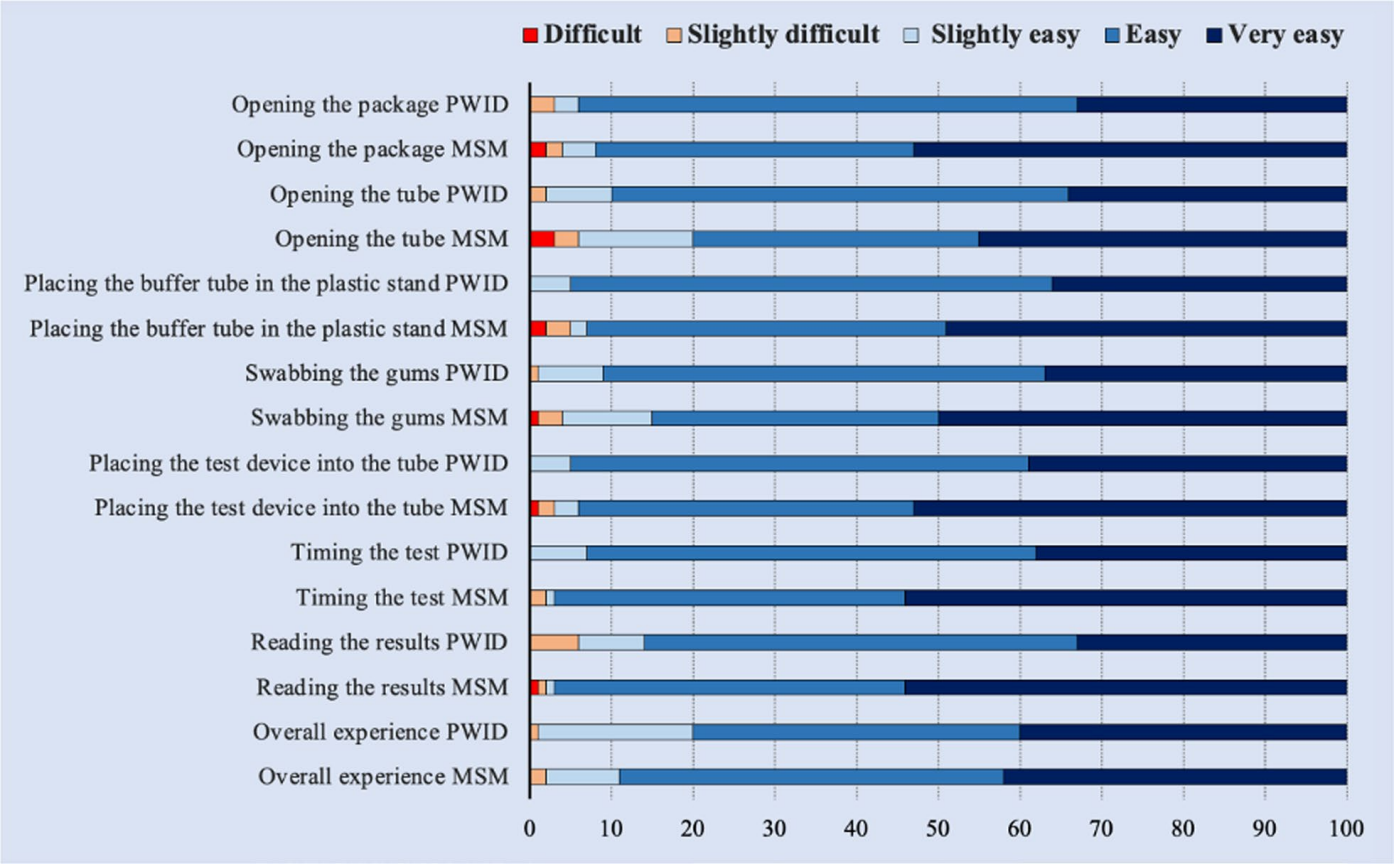
Ease of use rating of HCV self-testing steps among MSM/TG (n = 100) and PWID (n = 100)

The rating on the ease of use of the various HCVST steps by group is shown in Fig. 2 Overall, 80% of PWID and 89% of MSM/TG found the overall HCVST process easy or very easy to do. Compared to the PWID group, MSM/TG tended to rate all the testing steps as very easy.

#### Interpretation of HCV self-test results

Results for inter-reader and inter-operator agreement are summarized in Table 3. The HCV seropositivity rate, based on the results of the professional HCV test, was lower among MSM/TG group compared to PWID (2.2% vs 26.6%). The number of invalid results by the self-testers were 3 among PWID and 1 among MSM/TG. The overall inter-reader agreement was high among MSM/TG (98.9%) and PWID (96.7%). When paired invalid results were removed from the calculation, the inter-reader agreement among PWID and MSM remained unchanged. The overall inter-operator agreement was high both among MSM/TG (97.8%) as well as PWID (91.1%). Tests performed by the HCW using the professional HCV test yielded three additional positive cases among PWID (24 vs 21), which were missed both by the self-tester and the second reader, suggesting that a mistake in the self-testing procedure resulted in three false-negative results.

**Table 3:** OraQuick HCV Self-Test: inter-rater agreement by population group

Inter-reader agreement					Inter-operator agreement				
PWID	Re-reading by HCW				PWID	Re-testing by HCW			
Self-tester	Neg	Pos	Inv	Total	Self-tester	Neg	Pos	Inv	Total
Negative	65	2	0	67	Negative	63	4	0	67
Positive	1	19	0	20	Positive	1	19	0	20
Invalid	0	0	3	3	Invalid	2	1	0	3
Total	66	21	3	90	Total	66	24	0	90
Invalid rate self-tester: 3.33%					Invalid rate self-tester: 3.33%				
Agreement^1^: 96.7% Cohen’s kappa: 0.92					Agreement^1^: 91.1% Cohen’s kappa: 0.77				
Agreement^2^: 96.5% Cohen’s kappa: 0.90					Agreement^2^: NA				
MSM/TG	Re-reading by HCW				MSM/TG	Re-testing by HCW			
Self-tester	Neg	Pos	Inv	Total	Self-tester	Neg	Pos	Inv	Total
Negative	87	1	0	88	Negative	87	1	0	88
Positive	0	1	0	1	Positive	0	1	0	1
Invalid	0	0	1	1	Invalid	1	0	0	1
Total	87	2	1	90	Total	88	2	0	90
Invalid rate self-tester: 1.11%					Invalid rate self-tester: 1.11%				
Agreement^1^: 98.9% Cohen’s kappa: 0.80					Agreement^1^: 97.8% Cohen’s kappa: 0.49				
Agreement^2^: 98.9% Cohen’s kappa: 0.66					Agreement^2^: NA				

Agreement^1^: includes all test results

Agreement^2^: excludes paired invalid test results

#### Qualitative findings from cognitive interviews

There were three main areas where participants struggled with the test which were observed by the research assistants and then raised in the cognitive interviews: collecting the oral specimen; the interpretation of the test result; and timing and reading window of the test. These errors may have a negative impact on the accuracy of the test result.

##### Collecting the oral specimen

The correct collection of the oral fluid involves firmly pressing the pad against the gums and swabbing it along the upper and lower gum once. Five of the 20 participants had difficulties collecting the oral specimen. These were all from the PWID group and all of them had high school level education or lower. Two participants were unemployed and one was in a manual occupation. One participant only rubbed the pad on the bottom gum, another switched the side of the pad for each gum, and another participant only sampled halfway along each gum.

##### Interpretation of the result

More than half of the participants (11/20) struggled with some part of the interpretation of results. Many participants had difficulty understanding that a positive result meant they had antibodies to HCV and conversely that a negative result meant the antibodies were not detected. The word “positive” in the instructions was associated with a favourable outcome. In this case, some participants believed that a positive result meant they did not have HCV infection. Conversely, the word “negative” was associated with an unfavourable outcome, in this case, having HCV infection. Therefore, how HCV positive or negative results were translated in Georgian or worded in the instructions led to confusion on the true interpretation of the result by the participants.

“What does “positive” mean? It means that you don’t have virus, yes? … I think—I am confused, because I went for analyses, it did not show the virus and they say positive. This time I thought that I had virus, but it seems it was not and still they say positive. According to this, I think positive means that I don’t have (HCV). [112, PWID].

Some participants had difficulty understanding the difference between the control line and test line. They interpreted any single line as negative and therefore missing a possible invalid result. It was noted by some participants that there was no picture in the instructions depicting an invalid test that had a test line only, which may have affected some individual’s comprehension of the text. Some participants were not able to link the pictorial instructions of test device with results to the references of control and test lines in the text.

“Yes, it does not reflect the instruction, because there is different words in instruction, ok there is no line along C but there should be line along T, here is written that there is line along T. the red background if shown but there is not red line along T.” [104, PWID].

In the MSM/TG group, participants who struggled to interpret the different test results in the instructions were unemployed and only had a high school education, whereas those who successfully interpreted the test were mostly employed and had a university-level of education. In the PWID group, individuals who struggled with interpretation of test results also had a high school education or lower and were unemployed. Only one participant had a university-level of education and was able to interpret the different test results correctly.

##### Time requirement to ensure the correct reading window for the test

Half of participants (10/20) struggled to understand the instructions regarding the requirement to time the test and/or the need to read the results within the 20–40-min reading window. Some participants (6/20) did not understand why they needed a timer for the test and were confused by this:

“I don’t need them, I don’t need these devices, there are for measuring time, how long I will need to do the test, I don’t need them.” [105, PWID].

Often it was thought that a timer was required to measure how long the participant took to perform the test rather than how long to wait before reading the result. This was observed more commonly among PWID.

Thirteen participants did not understand the instruction regarding the manufacturer allowed time window that is required to read the test result. They were unclear as to what they were being asked to do, with the term “don’t read” being highlighted as difficult to understand, and not being viewed as connected to the interpretation of the test results. Many participants who did understand this instruction had to read this instruction carefully to understand the true intended meaning and identified it as hard to interpret.

“From the beginning it was difficult for me. …see, not read the results it is also needed to be mentioned, but if you read it, there is no sense, you cannot read them, if it does not become visible. If you change it and write that read your results after 20 min, it will be less confusing… And if it is directly written that put it inside and read it after 20 min, it will be more… in any case you will understand but still it is little difficult…” [108, PWID].

After inserting the test device in the developer fluid, nine out of 13 participants did not set the timer to time the test and had to be prompted by the researcher.

“… it is unclear, first they say read then don’t read, I cannot understand what they really want. Do they mean don’t look at it? … I don’t have watch or even the phone.” [107, PWID].

### Acceptability and preferences on HCVST

#### Quantitative findings

Table 4 provides a summary of the acceptability and preferences reported by PWID and MSM/TG. The pre-test acceptability was high in both populations, 98% in PWID and 96% in MSM/TG. After self-testing, the majority of PWID (91%) and MSM/TG (99%) would use it again and would take it to family or friends (95% and 94%). With respect to testing in the future, a higher proportion of MSM/TG would test at home compared to PWID (89% vs 73%; p = 0.004). A higher proportion of PWID preferred an oral-based test versus a blood-based test compared to MSM/TG (76% vs 56%; p = 0.001). The majority of MSM/TG (97%) said they would contact a health facility in case of a reactive test results while 47% of PWID would seek a confirmatory test. Knowledge about HCV treatment in both populations was generally high.

**Table 4:** Participant views and preference on HCVST

Population group	PWID (n = 100)		MSM/TG (n = 100)		*P* value
	No	%	No	%	
Pre-test acceptability					
Participation in the study	100/100	100.0	100/103	97.0	–
Would use HCVST if available	98	98.0	96	96.0	0.407
Post-test acceptability					
Would use OraQuick HCVST again	91	91.0	99	99.0	0.033
Would recommend HCVST to family/friends	95	95.0	97	97.0	0.758
Would take the test to family/friends	95	95.0	94	94.0	0.647
Preferences on HCVST					
Preferred approach to test HCV in future					
By myself at home	73	73.0	89	89.0	0.004
By myself at a health centre	0	0.0	38	38.0	< 0.001
In a health centre by a healthcare worker	12	12.0	16	16.0	0.221
In a screening campaign	6	6.0	4	4.0	0.516
Preferred sample type					
Oral fluid-based test	76	76.0	56	56.0	0.001
Blood-based test	10	10.0	6	6.0	
No preference	14	14.0	38	38.0	0.001
Steps taken if result of self-test is positive					
Contact healthcare facility	48	48.0	97	97.0	< 0.001
Contact pharmacy	0	0.0	9	9.0	0.002
Do a confirmatory test	47	47.0	5	5.0	< 0.001
Seek advice from family member/community	6	6.0	17	17.0	0.005
Do not know what to do	5	5.0	2	2.0	0.248
Knowledge about HCV treatment					
Know that HCV can be cured	76	76.0	63	63.0	0.063
Know there’s treatment but unsure about cure	19	19.0	35	35.0	–
No idea if there’s treatment or cure	5	5.0	2	2.0	–

#### Qualitative findings from in-depth interviews

The self-test was viewed by the majority of participants as easy to use. The test was seen as very convenient, especially the ability to test in a comfortable environment. Some participants trusted the result of a blood test more, as they were felt to be more accurate.

“The process of opening is wonderful. There are little cuts, from where you should tear it. A 2-year-old child could do that. Packing, everything is wonderful.” [004, MSM]

“...alone, at home. This test does not need doctor and professors, you will open it, do it, wait and see the result.” [113, PWID]

“I trust blood test more. But it is not absolute as well. The right way is to check you permanently—every 3 months.” [012, MSM]

There was massive support for HCVST to be universally available rather than targeted at certain key populations. Pharmacies were seen as the best route of distribution, though other routes such as through peers and workplace distribution were highlighted. The cost of the test was seen as important to uptake with an acceptable price point being 10 Georgian Lari ($3.50 USD).

“If we talk about target groups—it should be IDUs, but I think it also should be implemented for general population—as it is done abroad that they make checking every 6 months it will be great here—in Georgia as well” [117, PWID]

“It is better with peer educator, because when I saw that the test result on HIV was positive and the second line became visible, my curator started explaining that HIV is a manageable disease.” [011, MSM]

“I said it should be somewhere up to 10 GEL. If it will be cheaper it will be better.” [118, PWID]

Feelings related to stigmatisation of HCV such as fear, shame, and denial were cited by participants as psychological barriers to individuals accessing testing. This was linked to a lack of trust in the confidentiality of testing at both public and private facilities, especially in the MSM/TG group.

“You know what can be the reason, if they are employed in governmental structures its possible, they avoid testing because fear to lose the job, there is risk of it.” [008]

“...he will do it privately, confidentiality issues are solved with this self-test…” [003, MSM]

“The advantage of self-tests is using it anonymously; lots of community members does not want to be affiliated to community organizations and many people will order it online.” [012, MSM]

“There are lot of cases when people don’t do tests in laboratory, because they are afraid that somebody can see them in clinics and laboratories, it does not matter if person tests positive or negative and being seen in the clinics just that can be a stigma.” [011, MSM]

Most participants knew that HCV was a virus that affects the liver. Most participants understood the main routes of transmission, such as sexual contact and sharing needles, though a minority also had incorrect knowledge such as transmission via saliva and food.

“It is an infectious viral disease, if you find it early and have a treatment, it will be cured” [005, MSM]

“Hepatitis C is transmitted with medical and dental procedures, razors, sexual contact, with syringes, I mean drug use. Mostly it is common in PWIDs and in prisoners” [0005, PWID]

“As I know if you have some injury in your mouth and you drink from glass, with your saliva it will be transmitted” [004, MSM]

“Maybe it is transmitted through food, people are eating from trash bins, no?” [0001, MSM]

Most participants knew about the availability of treatment for HCV but many had anecdotal evidence of poor treatment outcomes or major side effects to treatment. Some acknowledged that this may be due to old treatment regimens. Further details about emerging topics and some quotations from participants participating in in-depth interviews can be found in Additional file 9.

“With quick test, with blood test as well and then you get treatment if you need to be included in Hepatitis C elimination program” [003, MSM]

“Hepatitis C can be cured with this medicine, but also many rumours or facts I don’t know, exist that say these medicines cause other health problems, like heart attack etc. We don’t know much about these medicines. It was not depended on Georgian doctors; it’s on the companies which gives medicines in Georgia…” [012, PWID]

“As I know the previous treatment—now they treat with pills and before I don’t know what it was—but it was terrible, because when they were telling me about it I became scared, it’s maybe because I was not doing test till now. Some said I had problems, some said I was absolutely disconnected for three months—they had negative feedback” [119, PWID]

## Discussion

Our study is one of the first to assess the usability and acceptability of HCVST among key populations in eastern Europe. Both the quantitative and qualitative data revealed that a high number of MSM/TG completed HCVST correctly and without assistance but it was significantly lower among PWID (80% vs 60%; p = 0.003). This was due to a higher occurrence of errors in at least one step of the testing process, 28.7% in PWID vs 16.7% in MSM/TG (p = 0.073). The PWID population in our assessment was older and with a lower educational level compared to the MSM/TG group, likely influencing the ability of participants to carry out HCVST correctly and independently.

Our findings are consistent with a similar twin study carried out in Vietnam [32]. Researchers found that the number of MSM conducting HCVST correctly was significantly higher compared to PWID (66.3% vs 37.1%; p < 0.0001), owing to sociodemographic differences among PWID such as being older and less educated. However, in the study in Georgia, we observed a much higher number of participants completing HCVST correctly, 83.3% in MSM vs 72.2% in PWID, although this difference was not statistically significant (p = 0.073). The higher usability found in our study may be explained by the fact that the Georgian PWID participants were much younger compared to those in Vietnam [median age (IQR): 35 years (28–41) vs 45 years (31–62)] and had a higher educational level with 30% of the PWID participants in Georgia holding at least a college degree vs 1% in Vietnam. Previous studies on HIVST have also found that older and less educated self-testers are more inclined to make mistakes during testing [16, 17]. A recent HCVST study in Kenya also found that PWID were more likely to make testing mistakes owing to participants experiencing withdrawal symptoms [39].

Inter-reader agreement was high among MSM/TG (98.9%; kappa = 0.80) and PWID (97.8%; kappa = 0.92), however, inter-operator agreement was slightly lower for PWID compared to MSM/TG (91.1% vs 96.7%). This reduced agreement was caused by 4 false-negatives and 1 invalid result reported by PWID, when in fact they were positive. A previous study evaluating the OraQuick HCV test in the USA also found that self-testers had difficulties reading the test lines and consequently reported false-negative results leading to a lower sensitivity among self-testers compared to trained staff (88.4% vs 97.3%) [28]. This is supported by comments in the qualitative interviews where participants highlighted misunderstandings about interpretation of different test lines and what positive and negative results meant.

Both quantitative and qualitative results combined revealed a high acceptability among MSM/TG and PWID in Georgia, matching those observed in earlier studies on HIVST showing a high acceptability and willingness to use HIVST among key populations [18, 19]. Perceived values and preferences as reported by MSM/TG and PWID are also similar to previous work on HIVST [40]. Although most participants expressed a preference for oral HCVST compared to blood-based HCVST, this was more pronounced among PWID where venous access may be challenging [27]. PWID found non-invasive oral testing more appealing as it is less painful than a finger stick. Previous studies on HIVST have shown varied preferences in sample type [40–43]. WHO guidelines on HCVST recommend different service delivery strategies including different type of self-test kits and specimens to increase uptake, and most importantly, that the varying preferences of different target populations are met [24]. Most participants considered HCVST easy to use and convenient. These results are consistent with a other studies conducted among MSM and PWID in the UK and Vietnam, which found HCVST to be easy to use and convenient [29, 32]. Waiting 20 min to read the test result was acceptable and is consistent with a facility-based study in Australia among PWID [44].

Although most participants knew about the availability of testing and treatment services in Georgia, some reinforced the need for additional information in the test package insert regarding what to do and where to go in case of a positive result. A previous study conducted among PWID and MSM in London also identified participants concerns about access to confirmatory testing and linkage to care and treatment [29]. Although innovations in diagnostics such as HCVST can contribute to bridging the gap in diagnosis, simplified and decentralised models of care are also urgently needed to achieve elimination targets [45, 46].

Our study has important implications for future work on HCVST. First, PWID faced more difficulties in successfully conducting HCVST compared to MSM/TG, and suggests that this group may require more direct assistance. Previous studies on HIVST have demonstrated that providing direct assistance or in-person demonstration to less-skilled users can increase the usability of self-tests [18, 47], so it is important that PWID are engaged and inform approaches and messaging that will address potential challenges around usability. Second, based on participants’ feedback there is a need to further optimize the instructions for use of the test. This includes translation of written instructions into local language; use of simple and clear language as some words proved difficult to interpret; include illustrations of all possible invalid results to aid in result interpretation, particularly when the test line is present but not the control line. Third, new HCVST studies using directly-assisted or video-assisted models in the PWID population are needed to inform areas for improvement of test usability, as well as studies evaluating potential distribution models and impact on testing uptake. Many lessons learned by HIVST during the COVID-19 pandemic should be leveraged to inform service delivery strategies and how to utilize virtual tools [48].

There are a number of caveats in the interpretation of findings from this study. First, the study sample size is small, and the study was conducted among MSM/TG and PWID in an urban area in Tbilisi, and results may therefore not be generalizable to other population groups and locations, such as rural areas. Second, this study was conducted in a country with a well-established HCV elimination programme. High awareness about the disease and access to confirmatory molecular testing and treatment in Georgia is likely to have contributed to higher levels of acceptability than in settings and populations without such a programme. Although well-trained interviewers conducted the quantitative and qualitative study, we cannot exclude some degree of social desirability bias in participants’ responses. However, in our study, qualitative findings matched those of the quantitative assessment, which minimizes the likely impact of this type of bias. Key strengths of this study were the use of a mixed-methods approach with triangulation between qualitative and qualitative findings, and further enriched with participants’ experiences and views.

## Conclusions

Taken together, our study shows that HCVST was highly acceptable among MSM/TG and PWID in Georgia. Better messaging and in-person demonstration as well as direct assistance to first-time self-testers or individuals with low literacy or education levels may be beneficial based on the findings among PWID. Despite notable progress of the Georgian HCV elimination programme, a substantial proportion of HCV-infected individuals remain undiagnosed. HCVST could be an additional approach to expand the coverage of HCV testing services and accelerate elimination efforts. Currently, HIVST is being introduced in the country among key populations [49], and this may serve as a strong foundation to also introduce HCVST.

## Supplementary Information

**Additional file 1.** OraQuick® HCV SELF-TEST KIT Instructions for Use (English).

**Additional file 2.** Baseine questionnaire.

**Additional file 3.** Product-specific checklist.

**Additional file 4.** Post-testing questionnaire.

**Additional file 5.** Cognitive interview guide.

**Additional file 6.** In-depth post-testing interview guide.

**Additional file 7.** Coding framework for qualitative data analysis.

**Additional file 8.** Sociodemographic characteristics of cognitive interview participants.

**Additional file 9.** In-depth post-testing interview topics and quotations.

## Data Availability

All data generated in the study are included in the article or uploaded as additional information. All data are fully available without restriction.

## Abbreviations

HCV: Hepatitis C virus
PWID: People who inject drugs
MSM: Men who have sex with men
TG: Transgender person
DAA: Direct-acting antiviral
WHO: World Health Organization
HIVST: HIV self-testing
HCVST: HCV self-testing
CI: Confidence interval
FIND: Foundation for innovative new diagnostics
LGBTQ +: Lesbian, gay, bisexual, transgender and queer people
Instructions: Instructions-for-use
HCW: Healthcare worker
IQR: Interquartile range
USD: United States Dollars
NGO: Non-governmental organization
