# Supplementary material for: Usability and acceptability of oral-based HCV self-testing among key populations: a mixed-methods evaluation in Tbilisi, Georgia
Source: BMC Infect Dis. 2022 May 31;22:510. doi: 10.1186/s12879-022-07484-2 (PMC9154030; doi:10.1186/s12879-022-07484-2)
Supplement: Supplementary file 1 — Additional file 1. OraQuick® HCV SELF-TEST KIT Instructions for Use (English). [file 12879_2022_7484_MOESM1_ESM.pdf]

## DIRECTIONS FOR USE

**You must follow the test directions carefully to get an accurate result.** Do not eat or drink for at least 15 minutes before you start the test or use mouth cleaning products 30 minutes before you start the test.

ENGLISH

# ORAQUICK® HCV SELF-TEST

## HOW TO USE THE OraQuick® HCV SELF-TEST KIT

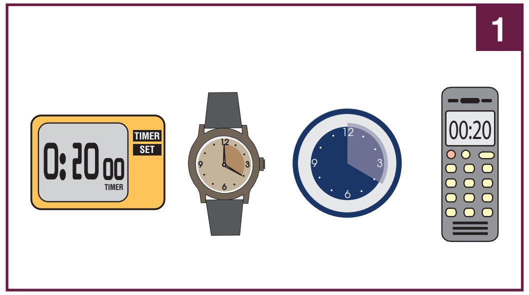


**YOU WILL NEED A WAY TO TIME THE TEST**

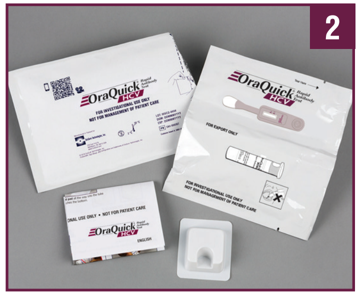


Pouch contains: **test kit, test stand and instructions.**

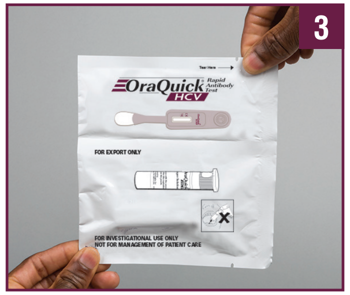


Your test kit contains two pouches.

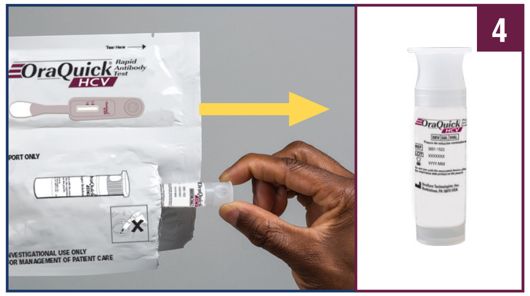


Tear open the pouch containing the **tube.**

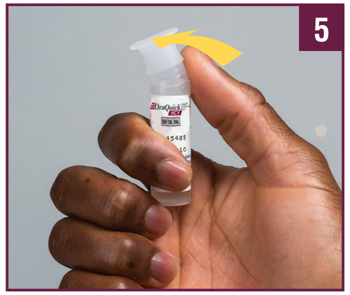


Remove the cap.

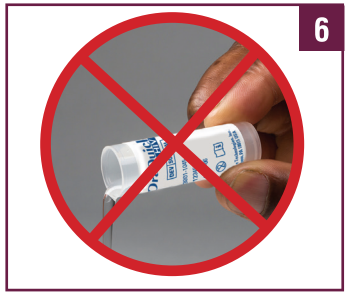


DO NOT pour out the **liquid.**

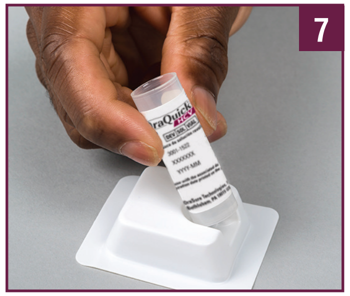


Slide the tube into the **stand.**

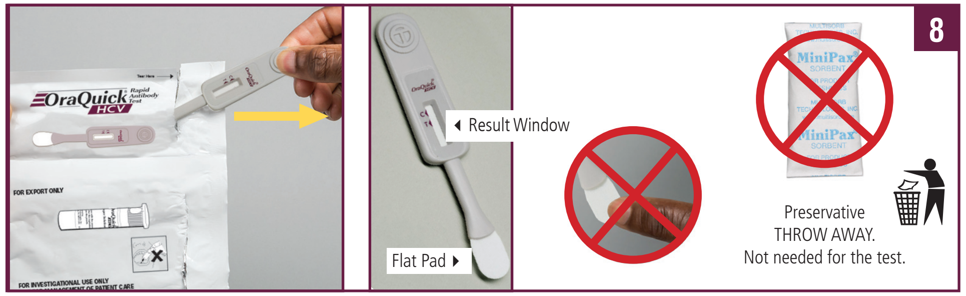


Tear open pouch containing the **test device** and remove. **DO NOT** touch the flat pad with your fingers.

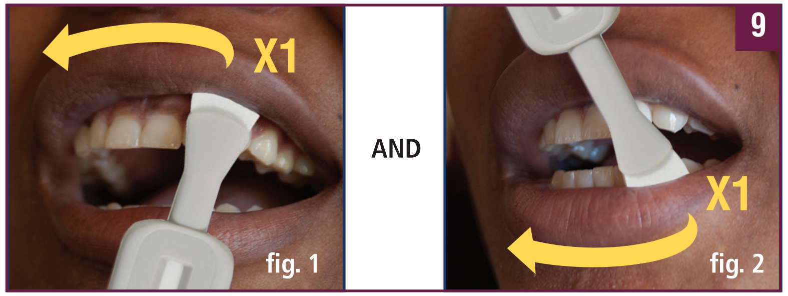


Press the **Flat Pad** firmly against your gum and swab it along your **upper gum once** (fig. 1) and **your lower gum once** (fig. 2).

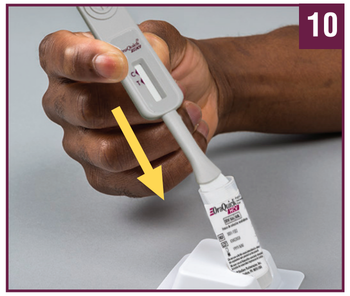


Slide the tube into the **stand.**

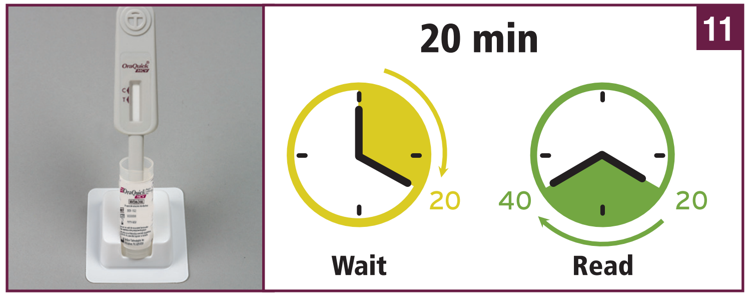


**LEAVE IT THERE for 20 minutes before reading the results.**
**DO NOT read the result after 40 minutes.**
**Test again in 6 months.**

**FOR INVESTIGATIONAL USE ONLY**
**NOT FOR MANAGEMENT OF PATIENT CARE**

**DRAFT**
rev. 01/19

# INTERPRETING RESULTS

## HCV POSITIVE RESULT

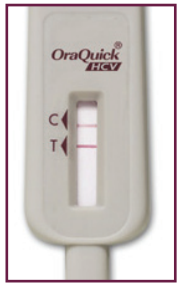

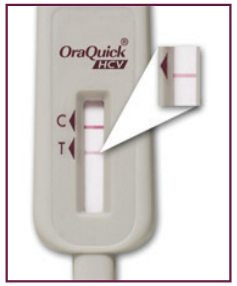


TWO LINES, even if the line is faint, means you may be HCV POSITIVE and you need to seek additional testing.

→

*As soon as possible . . .*

**Visit your nearest HCV Testing Center or Health Facility**

## HCV NEGATIVE RESULT

## IF READ BEFORE 20 MINUTES, RESULT MAY NOT BE CORRECT

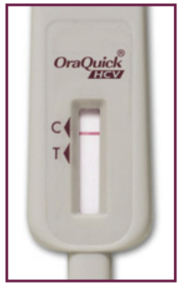


ONE LINE next to the "C" and NO line next to the "T", your result is HCV NEGATIVE.

→

Seek regular testing. If you may have been exposed to HCV test again in 3 months.

## INVALID RESULT

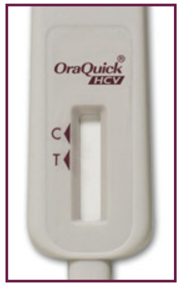

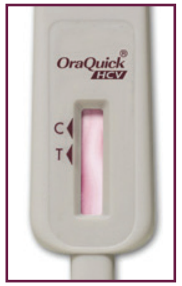


No line next to the "C" (even when there is a line next to the "T"), or a red background makes it impossible to read the test, the test is not working and should be repeated.

**You will need to obtain another test.**

→

The test did not work properly.

Visit your nearest HCV Testing Centre or Health Facility to test again.

## NOT SURE OF RESULT

You do not know your result or you are unsure of your result.

→

Visit your nearest HCV Testing Centre or Health Facility to test again.

# DISPOSE

Remove the test stick, put the cap on the test tube and throw away all contents in the normal trash.

Item# 3001-XXXX rev. 01/19

*Manufactured in Thailand for:* OraSure Technologies, Inc.
220 East First Street
Bethlehem, PA 18015
01+610-882-1820 • www.OraSure.com

© 2018 OraSure Technologies, Inc. • OraQuick®, logo design, and configuration are trademarks of OraSure Technologies, Inc.
