# Supplementary material for: Usability and acceptability of oral-based HCV self-testing among key populations: a mixed-methods evaluation in Tbilisi, Georgia
Source: BMC Infect Dis. 2022 May 31;22:510. doi: 10.1186/s12879-022-07484-2 (PMC9154030; doi:10.1186/s12879-022-07484-2)
Supplement: Supplementary file 2 — Additional file 2. Baseine questionnaire. [file 12879_2022_7484_MOESM2_ESM.docx]

**Supplement 2: BASELINE QUESTIONNAIRE**

**Usability and acceptability of oral-based HCV self-testing among key populations: A mixed-methods evaluation in Tbilisi, Georgia**

Emmanuel Fajardo, Victoria Watson, Moses Kumwenda, Dali Usharidze, Sophiko Gogochashvili, David Kakhaberi, Ana Giguashvili, Cheryl C Johnson, Muhammad S Jamil, Russell Dacombe, Ketevan Stvilia Philippa Easterbrook, Elena Ivanova Reipold.

| 1. **Demographic data of the participant** | | | |
| --- | --- | --- | --- |
| 1. **STUDY ID:**   *HC012-04-*  *__ __ __ __* | 1. **Date of testing:** *(dd/mmm/yy)*   -- | | 1. **Full name of the Interviewer:**   **__________________________**   1. **STUDY SITE**   **__________________________** |
| 1. **Sex at birth:**   🞎 Male 🞎 Female | 1. **Age:**   **___________**years | | 1. **City/Region of Provenance:**   🞎 Tbilisi  🞎Other:_____________________ |
| 1. **Marital status:**   🞎 Married or living with a partner  🞎 widow  🞎 divorced/separated (not living together)  🞎 Never married | 1. **Educational Background:**   *Indicate the highest educational level received*  🞎 Below high school  🞎 High school  🞎 College  🞎 Undergraduate (bachelor)  🞎 Postgraduate (master and above) | | |
| 1. **How many people live in the household?**   Total**:___**  Adults**:____**  Children**:____** | 1. **Main reason for your visit today?**   🞎 Screening for hepatitis 🞎 Screening for HIV/syphilis  🞎 Regular health check  🞎Other reason: __________________________  🞎 No answer | | |
|  | 1. **Main Occupation:** | | |
| 1. **HEPATITIS C TESTING AND MORBIDITIES** | | | |
| 1. **How often do you come to health facilities to check your health?**   🞎 More than 1 time per year  🞎 1 time per year  🞎 Rarely (once in 2 or more years)  🞎 Never | 1. **Please select all that apply. Have you have received or engaged in**   🞎 Unprotected anal intercourse  🞎 Injecting unprescribed drugs  🞎 Sharing needles  🞎 A surgical procedure 🞎 a dental procedure 🞎 Sharing shaving tools or toothbrushes  🞎 Make a tattoo  🞎 None of listed above | | 1. **Have you been tested for HCV?**   🞎 No, never.  🞎 Yes, more than 1 year ago  🞎 Yes, in the past 12 months  🞎 Do not know  🞎 Unwilling to disclose   1. **If yes, what was the results of the most recent testing?**   🞎 Positive 🞎 Negative 🞎 Don’t know |
| 1. **When was the last time you were tested for HIV? （If you were tested for HIV today, please tell us when was the previous time you were tested）**   🞎 I have never been tested for HIV  🞎 More than 1 year ago  🞎 In the past 12 months  🞎 Don’t know  🞎 Unwilling to disclose   1. **If yes, what was the results of the most recent testing?**   🞎 Positive 🞎 Negative 🞎 Don’t know | 1. **Have your partner been tested for HCV?**   🞎 No, never.  🞎 Yes, more than 1 year ago  🞎 Yes, in the past 12 months  🞎 Don’t know  🞎 Unwilling to disclose  🞎 No partner   1. **If yes, what was the results of the testing?**   🞎 Positive 🞎 Negative 🞎 Don’t know | | 1. **Have your partner been tested for HIV?**   🞎 No, never.  🞎 Yes, more than 1 year ago  🞎 Yes, in the past 12 months  🞎 Don’t know  🞎 Unwilling to disclose  🞎 No partner   1. **If yes, what was the results of the testing?**   🞎 Positive 🞎 Negative 🞎 Don’t know |
| 1. **Do you know that there are some tests that you can do at home to check for medical conditions (e.g. pregnancy test, glucose test)?**   🞎 No 🞎 Yes | | 1. **Have you ever used such a test (self-test) in the past?**   🞎 No 🞎 Yes, blood-based HIV self-test  🞎 Yes, other: _____________________ | |
| 1. **If such a test was available to check for hepatitis C, would you be willing to use it?**   🞎 No 🞎 Yes | | | |
| **Additional Observations/Comments:** | | | |
