# Supplementary material for: Usability and acceptability of oral-based HCV self-testing among key populations: a mixed-methods evaluation in Tbilisi, Georgia
Source: BMC Infect Dis. 2022 May 31;22:510. doi: 10.1186/s12879-022-07484-2 (PMC9154030; doi:10.1186/s12879-022-07484-2)
Supplement: Supplementary file 3 — Additional file 3. Product-specific checklist. [file 12879_2022_7484_MOESM3_ESM.docx]

**Supplement 3: CHECKLIST ON SELF-TESTING PROCESS**

**Usability and acceptability of oral-based HCV self-testing among key populations: A mixed-methods evaluation in Tbilisi, Georgia**

Emmanuel Fajardo, Victoria Watson, Moses Kumwenda, Dali Usharidze, Sophiko Gogochashvili, David Kakhaberi, Ana Giguashvili, Cheryl C Johnson, Muhammad S Jamil, Russell Dacombe, Ketevan Stvilia Philippa Easterbrook, Elena Ivanova Reipold.

| **Review during THE proceedure** |
| --- |
| **Name of the reviewer:** |
| \| 1. **CHECKLIST** \| \| \| --- \| --- \| \| 1. Did the study participant open the pouch and take all of the contents out? \| 🞏 Yes 🞏No  If no, why: _________________________ \| \| 1. Did the study participant read/use the instructions for use before the testing? \| 🞏 Yes 🞏No  If no, why: _________________________ \| \| 1. Did the study participant read/use the instructions for use during the testing? \| 🞏 Yes 🞏No  If no, why: _________________________ \| \| 1. Did the study participant to remove the test tube from the test pack? \| 🞏 Yes 🞏No  If no, why: _________________________ \| \| 1. Did the study participant remove the cap from the test tube? \| 🞏 Yes 🞏No  If no, why: _________________________ \| \| 1. Did the study participant place the test tube in the test stand? \| 🞏 Yes 🞏No  If no, why: _________________________ \| \| 1. Was the study participant able to remove the test device from the test pack? \| 🞏 Yes 🞏No  If no, why: _________________________ \| \| 1. Did the study participant touch the flat pad? \| 🞏 Yes 🞏No  If yes, why: _________________________ \| \| 1. Did the study participant collect the sample correctly (1x upper and 1x lower swab of gums)? \| 🞏 Yes 🞏No  If no, why: _________________________ \| \| 1. Did the study participant place the test device in the test tube correctly? \| 🞏 Yes 🞏No  If no, why: _________________________ \| \| 1. Did the study participant use a time keeping device (Clock, watch, timer)? \| 🞏 Yes 🞏No  If no, why: _________________________ \| \| 1. Did the study participant read the device results between 20 and 40 minutes after placing the device in the test tube? \| 🞏 Yes 🞏No  If no, why: _________________________ \| |
| 1. **ADDITIONAL DESCRIPTION DURING THE TEST** |
| **1.**  **Mark ALL the problems or mistakes observed when performing the test** |
| 🞎 Difficulties with opening the pouch  🞎 Touched the flat pad  🞎 Rubbed the wrong part of the mouth  🞎 Rubbed only upper or lower gums and not both  🞎 Spilt the fluid from the tube  🞎 Poured the fluid from the tube into the stand  🞎 Difficulties with sliding the tube into the stand  🞎 Test device came out of the tube while testing  🞎 Test results read before time  🞎 Not able to identify the test line or the control line  🞎 Interpreted test results wrongly  🞎 No problems or mistakes observed  🞎 Any other  Specify:___________________________________________________________________________________ |
| **2. Which part of self-testing you observed was the most difficult to perform? (Mark one or two)** |
| 🞎 Opening the pouch  🞎 Opening the tube  🞎 Swabbing the mouth  🞎 Sliding the tube into the stand  🞎 Placing the test device into the tube  🞎 Reading the results  🞎 All the steps were easy  🞎 All the steps were difficult |
| **3. Was the testing procedure completed?** |
| 🞎 Yes 🞎 No |
| **4. Was assistance provided? If yes, at which step:** |
| 🞎 No  🞎 Yes, opening the pouch  🞎 Yes, opening the tube  🞎 Yes, sliding the tube into the stand  🞎 Yes, placing the test device into the tube  🞎 Yes, reminding the test incubation time  🞎 Yes, reading the results  🞎 Any other  Specify:___________________________________________________________________________________ |
