# Supplementary material for: Usability and acceptability of oral-based HCV self-testing among key populations: a mixed-methods evaluation in Tbilisi, Georgia
Source: BMC Infect Dis. 2022 May 31;22:510. doi: 10.1186/s12879-022-07484-2 (PMC9154030; doi:10.1186/s12879-022-07484-2)
Supplement: Supplementary file 4 — Additional file 4. Post-testing questionnaire. [file 12879_2022_7484_MOESM4_ESM.docx]

**Supplement 4: POST-TESTING QUESTIONNAIRE**

**Usability and acceptability of oral-based HCV self-testing among key populations: A mixed-methods evaluation in Tbilisi, Georgia**

Emmanuel Fajardo, Victoria Watson, Moses Kumwenda, Dali Usharidze, Sophiko Gogochashvili, David Kakhaberi, Ana Giguashvili, Cheryl C Johnson, Muhammad S Jamil, Russell Dacombe, Ketevan Stvilia Philippa Easterbrook, Elena Ivanova Reipold.

*Thank you for agreeing to participate today and give your informed consent. I would like to ask you about your experiences of oral self-assessment for HCV self-testing. All your answers will remain confidential and you do not have to answer to questions that you do not want. There are no rights or wrong answers to these questions. Please free to ask questions anytime during the interview and we can stop at any time. Thank you again for your participation.*

| **Acceptability and feasibility of HCV self-testing** |
| --- |
| **Name of the reviewer:** |
| **How easy or difficult was it to understand the written instructions for use? *Read the options*** |
| 🞎 Very easy 🞎 Somewhat easy 🞎 Somewhat difficult 🞎 Very difficult 🞎 Not used |
| **2. How useful were written instruction during the testing procedure?  *Read the options*** |
| 🞎 Very much 🞎 Somewhat 🞎 A little 🞎 Not at all |
| **3. Thinking about the test, how did you find performing each of these steps:  *Read the options*** |
| \|  \| **Very difficult** \| **Difficult** \| **Slightly difficult** \| **Slightly easy** \| **Easy** \| **Very easy** \| \| --- \| --- \| --- \| --- \| --- \| --- \| --- \| \| **Opening the package** \| ⃝ \| ⃝ \| ⃝ \| ⃝ \| ⃝ \| ⃝ \| \| **Opening the tube** \| ⃝ \| ⃝ \| ⃝ \| ⃝ \| ⃝ \| ⃝ \| \| **Sliding the tube into the stand** \| ⃝ \| ⃝ \| ⃝ \| ⃝ \| ⃝ \| ⃝ \| \| **Swabbing the gums** \| ⃝ \| ⃝ \| ⃝ \| ⃝ \| ⃝ \| ⃝ \| \| **Placing the device into the tube** \| ⃝ \| ⃝ \| ⃝ \| ⃝ \| ⃝ \| ⃝ \| \| **Timing the test** \| ⃝ \| ⃝ \| ⃝ \| ⃝ \| ⃝ \| ⃝ \| \| **Reading the results** \| ⃝ \| ⃝ \| ⃝ \| ⃝ \| ⃝ \| ⃝ \| |
| **4. Please rate your overall experience with self-test:  *Read the options*** |
| 🞎 Very easy 🞎 Easy 🞎 Slightly easy 🞎 Slightly difficult 🞎 Difficult 🞎 Very difficult |
| **5. In your opinion, is HCV ST an accurate test? (Do you trust these results?)  *Read the options*** |
| 🞎 Very accurate 🞎 Somewhat accurate 🞎 Not accurate 🞎 Do not know |

| **6. Overall, how satisfied were you with the HCV self-testing process? *Read the options*** |
| --- |
| 🞎 Very satisfied 🞎 Somewhat satisfied 🞎 A little satisfied 🞎 Not satisfied at all |
| 1. **Would you recommend HCV self-testing to your partner, friend or family member?** |
| 🞎 Yes 🞎 No 🞎 Not sure  **Why?**  ________________________________________________________________________________________ |
| 1. **How would you feel about taking tests home for your partner, friend or family member?** |
| 🞎 Positive 🞎 Prefer not to 🞎 Not sure  **Why?**  ________________________________________________________________________________________ |
| 1. **Would you like to use this hepatitis C self-test again?** |
| 🞎 Yes 🞎 No 🞎 Not sure |
| 1. **In your opinion, what are the advantages of self-testing for hepatitis C?** |
| 🞎 The test can be performed in privacy; 🞎 No need to come to a clinic; 🞎 I can test myself anytime;  🞎 Not sure; 🞎 None; 🞎 Other, specify:______________________________________________________ |
| 1. **Which are the disadvantages of self-testing for hepatitis C?** |
| 🞎 Difficult to perform; 🞎 No confidence in test results; 🞎 Getting the results alone (no counselling);  🞎 Need to pay for the test; 🞎 Not sure; 🞎 None; 🞎 Other, specify:_______________________________  _______________________________________________________________________________________ |
| 1. **What would be your preferred mode to test for Hepatitis C in the future? *Read the options*** |
| 🞎 By myself at home; 🞎 By myself at a healthcare facility; 🞎 In a healthcare facility by Healthcare Worker;  🞎 In a screening campaign; 🞎 Taking a regular sample at a healthcare facility;  🞎 Any, No specific preference; 🞎 Not sure |
| 1. **Which way would you prefer to self-test: using oral fluids or fingerstick blood?** |
| 🞎 Oral fluids; 🞎 Fingerstick blood; 🞎 No preference  **Why?**  ______________________________________________________________________________________ |
| 1. **In the case of doing the test by yourself, would you be comfortable on reading any result alone?** |
| 🞎 Yes 🞎 No 🞎 Not sure  **Why?**  ______________________________________________________________________________________ |
| 1. **What would you do if your HCV self-test is giving a positive result (indicating a likelihood of having hepatitis C infection)? *Check all that applies*** |
| 🞎 Contact health facility; 🞎 Contact pharmacy; 🞎 Do a confirmation test (viral load test);  🞎 Seek advice from family members and/or friends;  🞎 Seek advice from a community representative (e.g. NGO representative); 🞎 Do not know  🞎 Other: please specify: ____________________________________________________________ |
| 1. **Do you know if people can be treated and cured for Hepatitis C?** |
| 🞎 Yes, there is a treatment, but not sure about cure 🞎 Yes, there is treatment and cure  🞎 Not sure if treatment or cure 🞎 There is no treatment or cure 🞎 No idea |
| 1. **Do you know if there is treatment available for Hepatitis C in your village/town or near your village/town?** |
| 🞎 Yes 🞎 Yes, but not nearby 🞎 No 🞎 Not sure 🞎 No idea |
| 1. **Interviewer comments on specific questions, respondent, interview, any additional** |
|  |

**Thank you for your participation. Do you have any questions you would like to ask?**
