# Supplementary material for: Usability and acceptability of oral-based HCV self-testing among key populations: a mixed-methods evaluation in Tbilisi, Georgia
Source: BMC Infect Dis. 2022 May 31;22:510. doi: 10.1186/s12879-022-07484-2 (PMC9154030; doi:10.1186/s12879-022-07484-2)
Supplement: Supplementary file 5 — Additional file 5. Cognitive interview guide. [file 12879_2022_7484_MOESM5_ESM.docx]

**Supplement 5. COGNITIVE INTERVIEW GUIDE: HCV self-testing among MSM/TG and PWID**

**Usability and acceptability of oral-based HCV self-testing among key populations: A mixed-methods evaluation in Tbilisi, Georgia**

Emmanuel Fajardo, Victoria Watson, Moses Kumwenda, Dali Usharidze, Sophiko Gogochashvili, David Kakhaberi, Ana Giguashvili, Cheryl C Johnson, Muhammad S Jamil, Russell Dacombe, Ketevan Stvilia Philippa Easterbrook, Elena Ivanova Reipold.

**Opening statements:**

**Thank you for taking time to speak with me. As you may remember, my name is _____. We have asked you to participate in this exercise because we are studying the feasibility of HCV self-testing in Georgia. For people to accurately and safely self-test, they should have clear instructions. We are aiming to develop clear instructions that can be used for HCV self-testing. As discussed during the consent discussion, we will ask you to self-test using instructions we have developed. As you go through the process, we will ask you questions about your understanding of the instructions. This process is likely to take about one hour to complete. Feel free to let me know if you need a break at any time. You can also stop the interview if you do not want to continue the discussion. Before we begin do you have any questions?**

**PLEASE TAKE A LOOK AT THE SHEET THAT EXPLAINS THE INSTRUCTIONS FOR SELF-TESTING.**

**General**

1. What do you think of the layout of the instructions?

*Probe: Do you understand where to start and which order of the instructions to follow?*

1. What information is contained on the first page? What information is contained on the second page?

**Heading**

1. What is your understanding of the instructions on the top of the first page?
2. What is your understanding of the warning on the top of the first page (with the image of the triangle)?

**Instruction number 1 - need for a timing device**

1. Please tell me in your own words what instruction number 1 is telling you.

*Probes: How clear is the image? How clear is the instruction? How could we modify the image or reword it to make it clearer? Do you think another person would understand what is meant by “way to time the test”?*

**Instruction number 2 - contents of package**

1. Please tell me in your own words what instruction number 2 is telling you.

*Probes: How clear is the image? How clear is the instruction? How could we modify the image or reword it to make it clearer?*

**Instruction number 3 - characteristics of package**

1. Please tell me in your own words what instruction number 3 is telling you.

*Probes: How clear is the image? How clear is the instruction? How could we modify the image or reword it to make it clearer? Do you think another person would understand what is meant by “two pouches”?*

**Instruction number 4 - removal of tube from pouch**

1. Please tell me in your own words what instruction number 4 is telling you to do.

*Probes: How clear is the image? How clear is the instruction? How could we modify the image or reword it to make it clearer? Do you think another person would understand what is meant by “pouch containing the tube”?*

1. Please go ahead and carry out the instruction as you understand it. [RECORD OBSERVATIONS]

**Instruction number 5 - opening of tube**

1. Please tell me in your own words what instruction number 5 is telling you to do.

*Probes: How clear is the image? How clear is the instruction? How could we modify the image or reword it to make it clearer? Do you think another person would understand what is meant by “remove the cap”?*

1. Please go ahead and carry out the instruction as you understand it. [RECORD OBSERVATIONS]
2. How easy or difficult was it to remove the cap of the tube?

*Probes: If there were any problems, how could these be resolved?*

**Instruction number 6 - not pouring the liquid in the tube**

1. Please tell me in your own words what instruction number 6 is telling you to do.

*Probes: How clear is the image? How clear is the instruction? How could we modify the image or reword it to make it clearer? Do you think another person would understand what is meant by “DO NOT pour out the* ***liquid”****?*

**Instruction number 7 - sliding of tube into stand**

1. Please tell me in your own words what instruction number 7 is telling you to do.

*Probes: How clear is the image? How clear is the instruction? How could we modify the image or reword it to make it clearer? Do you think another person would understand what is meant by “slide the tube into the stand”?*

1. Please go ahead and carry out the instruction as you understand it. [RECORD OBSERVATIONS]
2. How easy or difficult was it to slide the tube into the stand?

*Probes: Were there any problems identifying the stand? Did any liquid spill as you placed the open vial into the stand? If there were any problems, how could these be resolved?*

**Instruction number 8 - removal of test device from pouch**

1. Please tell me in your own words what instruction number f8 is telling you to do.

*Probes: How clear is the image? How clear is the instruction? How could we modify the image or reword it to make it clearer? Do you think another person would understand what is meant by the “test device” and “flat pad”?*

1. Please go ahead and carry out the instruction as you understand it. [RECORD OBSERVATIONS]
2. How easy or difficult was it to open the pouch and remove the test device?

*Probes: Were there any problems identifying which part was the flat pad and which part was the results window? Did you accidentally touch the flat pad? Did you understand what to do with the preservative? If there were any problems, how could these be resolved?*

**Instruction number 9 - collection of specimens**

1. Please tell me in your own words what instruction number 9 is telling you to do.

*Probes: How clear is the image? How clear is the instruction? How could we modify the image or reword it to make it clearer? Do you think another person would understand what is meant by the “press the flat pad against your gum” and “swab it along your upper gum/lower gum”?*

1. Please go ahead and carry out the instruction as you understand it. [RECORD OBSERVATIONS]
2. How easy or difficult was it to collect the specimen?

*Probes: If there were any problems, how could these be resolved?*

**Instruction number 10 - placement of test device**

1. Please tell me in your own words what instruction number 10 is telling you to do.

*Probes: How clear is the image? How clear is the instruction? How could we modify the image or reword it to make it clearer?*

1. Please go ahead and carry out the instruction as you understand it. [RECORD OBSERVATIONS]
2. How easy or difficult was it to put the test device into the tube?

*Probes: If there were any problems, how could these be resolved?*

**Instruction number 11 - timing of test**

1. Please tell me in your words what instruction number 11 is telling you to do?

*Probes: How clear is the image? How clear is the instruction? How could we modify the image or reword it to make it clearer? What may happen if you read your result early, or if you read your result after 40 minutes? What can be done if one does not have a watch?*

**Interpreting results - HCV reactive**

1. Please tell me in your own words what a reactive result looks like.

*Probes: How clear is the image? How clear is the instruction? How could we modify the image or reword it to make it clearer? Do you think another person would be able to understand the instructions and read their reactive result correctly?*

1. Please tell me in your own words what should be done if one test is HCV reactive.

*Probes: How clear is the image? How clear is the instruction? How could we modify the image or reword it to make it clearer? Do you think another person would be able to understand what should be done after a reactive result?*

**Interpreting results - HCV negative**

1. Please tell me in your own words what a negative result looks like.

*Probes: How clear is the image? How clear is the instruction? How could we modify the image or reword it to make it clearer? Do you think another person would be able to understand the instructions and read their negative result correctly?*

1. Please tell me in your own words what should be done if one tests HCV negative.

*Probes: How clear is the image? How clear is the instruction? How could we modify the image or reword it to make it clearer? Do you think another person would be able to understand what should be done after a negative result?*

**Interpreting results - invalid**

1. Please tell me in your own words what an invalid result looks like.

*Probes: How clear is the image? How clear is the instruction? How could we modify the image or reword it to make it clearer? Do you think another person would be able to understand the instructions and read their invalid result correctly?*

1. Please tell me in your own words what should be done if one gets an invalid result.

*Probes: How clear is the image? How clear is the instruction? How could we modify the image or reword it to make it clearer? Do you think another person would be able to understand what should be done after an invalid result?*

**Interpreting results - not sure of the result**

1. Please tell me in your own words what should be done if one is not sure about self-test result.

*Probes: How clear is the instruction? How could we reword it to make it clearer? Do you think another person would be able to understand what should be done after failing to understand the meaning of their result?*

**Interpreting own results**

1. Please read your results. [RECORD OBSERVATIONS]
2. How easy or difficult was it to interpret your results?

*Probes: Were you able to relate the appearance of your test to any of the pictures in the instructions?* *If there were any problems, how could these be resolved?*

1. Do you feel confident that this result is correct?

*Probes: Do you think another person would believe the result? Why? If no, what could be done so that people have confidence in the test result?*

1. Is there any other information that you would like to have about what to do after taking the HCV self-test?

**Test kit disposal**

1. Please describe in your own words what to do with the contents of the test kit after the self-test?

*Probes: How clear is the image? How clear is the instruction? How could we modify the image or reword it to make it clearer? Do you think another person would be able to understand the instructions and what to do with the contents of the test kit?*
