# Supplementary material for: Usability and acceptability of oral-based HCV self-testing among key populations: a mixed-methods evaluation in Tbilisi, Georgia
Source: BMC Infect Dis. 2022 May 31;22:510. doi: 10.1186/s12879-022-07484-2 (PMC9154030; doi:10.1186/s12879-022-07484-2)
Supplement: Supplementary file 6 — Additional file 6. In-depth post-testing interview guide. [file 12879_2022_7484_MOESM6_ESM.docx]

**Supplement 6. IN DEPTH INTERVIEW GUIDE: HCV self-testing among MSM/TG and PWID**

**Usability and acceptability of oral-based HCV self-testing among key populations: A mixed-methods evaluation in Tbilisi, Georgia**

Emmanuel Fajardo, Victoria Watson, Moses Kumwenda, Dali Usharidze, Sophiko Gogochashvili, David Kakhaberi, Ana Giguashvili, Cheryl C Johnson, Muhammad S Jamil, Russell Dacombe, Ketevan Stvilia Philippa Easterbrook, Elena Ivanova Reipold.

### Opening statements:

Thank you for agreeing to take part in this interview today. We will be discussing your experiences with HCV testing and your views on how HCV self-testing should be provided.

1. **Current HCV testing**
2. Can you please tell us about what you know about hepatitis C?

- *PROBE*: what is it? How is it transmitted? Who are the people that are infected in your community? How is it tested for? How is it treated?

1. How is hepatitis C viewed in the community?

- *PROBE: Is it viewed positively or negatively? Why? In the general population? In your specific group (MSM, PWID)? How does it compare to HIV?*

1. Under what circumstances, if any, should people be testing for HCV?

- *PROBE: Do you know what the risk factors are? Do you consider yourself at risk? sources of information on HCV*

1. What services are currently available for HCV testing?

- PROBE: What current options for HCV testing are available to you? *Briefly discuss the advantages and disadvantages of each option. Focus on access: location, transportation, quality of staff and treatment of clients, cost of services, time the services are provided etc.*

1. What do you think are the main reasons why people choose to go for HCV testing?

*PROBE: Which groups of people usually go for HCV testing? (i.e. age, sex, economic status). What about the main reasons why women go for HCV testing? Men? Adolescents? Adults 40 years or older? MSM? IVDUs? Are these reasons specific to HCV testing in general, ~~or facility-based testing only~~?*

1. What do you think are the main reasons why people don’t go for HCV testing?

- *PROBE: Which groups of people who are at risk of hepatitis infection do not usually go for HCV testing? (i.e. age, sex, economic status). What about the main reasons why women don’t go for HCV testing? Men? Adolescents? Adults 40 years or older? IVDUs? MSM? Are these reasons specific to HCV testing in general, ~~or facility-based testing only~~?*

1. How do people access treatment for HCV?

- PROBE: Do they know where to go? What is easy about accessing treatment? What is difficult? What are the benefits of receiving treatment? What are the disadvantages of receiving treatment?

1. **HCV self-testing**
2. What do you think about allowing people to self-test for hepatitis C?

- PROBE: What do you think are the advantages and disadvantages?

1. What do you think of this HCV self-testing kit?

- *PROBE: What were your experiences in using it? What did you like or dislike about this test kit? What was easy about using the kit? What did you find difficult? Clarity of word and pictorial instructions? Taking the specimen? Ease of performance? Clarity of reading results? Packaging of the kit? Presentation and user friendliness of the kit? Which areas should be improved to make it easier for people to test themselves?*

1. Would you use an HCV oral self-test again in the future?

- *PROBE: Did you feel confident in the result you gained? Would you prefer a blood-based self- test? Why or why not?*

1. Do you think other people would be interested in HCV self-testing? Why or why not?

- *PROBE: Who do you think would benefit most from HCV self-testing in your community? Do you think it would be preferred over facility-based testing? Do you think it would increase the frequency of when people test? What specific conditions would need to be in place for individuals and the community to accept HCV self-testing? If HCV self-test were to be sold, what price would be acceptable to you?*

1. If HCV self-testing were to become available, how do you think it should be provided?

- *PROBE: Where and when should people be able to access the HCV self-testing kits? What price would be acceptable for HCV self-test? How should support for linkage to confirmatory HCV testing and treatment be provided? What about linkage to confirmatory HCV testing and treatment specific to injecting drug users or men who have sex with men? Which groups of people require more support and why? How long would people usually take to go to a clinic after a positive test result?*

1. Would you prefer to test at home by yourself, with your friends or with assistance of the healthcare worker (or peer)?

- *PROBE: Why? What information/counselling should be given? When and how should it be given (pre-/post-test)?*
- *What are the safety concerns that are linked to HCV self-testing? What would be the best way to anticipate and reduce the likelihood of safety issues?*

1. If HCV self-testing were to become available*,* who should have access to HCV self-testing kits?

- PROBE: Are *there specific people or groups who should be prioritized? Explain.*
- *Would you be comfortable taking the test to your partner/friends? In what why do you think it would affect your relationships?*

Thank you for your time and participation. We have learnt a lot from our discussion here today and we hope the time has also been useful to you.

Do you have any questions for me?
