# Supplementary material for: Usability and acceptability of oral-based HCV self-testing among key populations: a mixed-methods evaluation in Tbilisi, Georgia
Source: BMC Infect Dis. 2022 May 31;22:510. doi: 10.1186/s12879-022-07484-2 (PMC9154030; doi:10.1186/s12879-022-07484-2)
Supplement: Supplementary file 7 — Additional file 7. Coding framework for qualitative data analysis. [file 12879_2022_7484_MOESM7_ESM.docx]

**Supplement 7: Coding framework for in-depth interviews on HCV Testing**

**Usability and acceptability of oral-based HCV self-testing among key populations: A mixed-methods evaluation in Tbilisi, Georgia**

Emmanuel Fajardo, Victoria Watson, Moses Kumwenda, Dali Usharidze, Sophiko Gogochashvili, David Kakhaberi, Ana Giguashvili, Cheryl C Johnson, Muhammad S Jamil, Russell Dacombe, Ketevan Stvilia Philippa Easterbrook, Elena Ivanova Reipold.

| **Routine HCV Testing** | **HCV Self- testing** | **HCV Treatment** | **HCV knowledge** |
| --- | --- | --- | --- |
| Psychological  Financial  Time  Delay  Accuracy  Sample  Addiction  Access | Acceptability  Financial  Sample  Usability  Interpretation  Instructions for use  Areas for improvement  Confidentiality  Additional support  Linkage to care  Distribution of HCV self-testing | Access  Cost  Side effects  Readiness | Source  Test access  Transmission  HCV testing  HCV treatment  Disease  Test groups  Community perceptions of HCV infection |

**Code Definitions**

**Routine HCV testing**

Psychological - *State of mind issues that will stop people accessing routine services or getting tested (e.g. fear, laziness)*

Financial - *Related to cost of both test and access to it*

Time - *Time taken to access testing services*

Delay - *Time between accessing test and getting result*

Accuracy - *How accurate should the test be and what effect does this have on the individual*

Sample - *Sample taken and the effect on the user*

Addiction - *Impact of addiction on testing access and performance*

Access to testing - *Views on access to testing services*

**HCV Self-testing**

Acceptability - *Use again, recommend the test? Advantages and disadvantages, Confidence in result- trust in results, safety concerns*

Financial - *Cost of the HCV self-test, how much willing to pay for a HCV self-test*

Sample type - *Blood versus oral*

Usability - *How easy was the test to perform*

Interpretation of results- *Were they able to interpret the test result/confidence in result*

Instructions for use - *Pictures, wording, understanding*

Areas for improvement - *Ideas how the test could be improved*

Confidentiality - *Is confidentiality important*

Additional support - *Counselling, help during the testing process*

Linkage to care - *What to do/where to go, time to access*

Distribution of HCV self-test - *Additional support for any groups; Provision to a friend; Where to distribute; Who should have access (priority groups); Whom to test with*

**Treatment**

Access – *Issues of access to HCV treatment*

Cost – *Cost of HCV treatment and effect on access/uptake/continuation*

Side effects – *Knowledge of and effect of side effects*

Readiness – *What is required of an individual to undertake treatment*

**HCV knowledge**

Source of information – *Where do they get their knowledge from*

Test Access – *Where can they get tested. Why do they get tested*

Transmission – *How is HCV transmitted*

HCV testing – *How is the testing done*

HCV treatment – *How is HCV treated*

Disease – *What do they know about the disease*

Test groups – *Why and who should test, male, female, PWIDs*

Community perceptions - *of HCV infection; PWIDs attitudes to HCV; what is the community general knowledge of HCV*
