# Supplementary material for: Usability and acceptability of oral-based HCV self-testing among key populations: a mixed-methods evaluation in Tbilisi, Georgia
Source: BMC Infect Dis. 2022 May 31;22:510. doi: 10.1186/s12879-022-07484-2 (PMC9154030; doi:10.1186/s12879-022-07484-2)
Supplement: Supplementary file 8 — Additional file 8. Sociodemographic characteristics of cognitive interview participants. [file 12879_2022_7484_MOESM8_ESM.docx]

**Supplement 8: SOCIODEMOGRAPHIC CHARACTERISTICS OF INDIVIDUALS PARTICIPATING IN COGNITIVE AND IN-DEPTH INTERVIEWS**

**Usability and acceptability of oral-based HCV self-testing among key populations: A mixed-methods evaluation in Tbilisi, Georgia**

Emmanuel Fajardo, Victoria Watson, Moses Kumwenda, Dali Usharidze, Sophiko Gogochashvili, David Kakhaberi, Ana Giguashvili, Cheryl C Johnson, Muhammad S Jamil, Russell Dacombe, Ketevan Stvilia Philippa Easterbrook, Elena Ivanova Reipold.

| **Supplement 8** Sociodemographic characteristics of a subset of participants | | | | | |
| --- | --- | --- | --- | --- | --- |
| **Qualitative method** | **Cognitive Interview** | |  | **In-depth Interview** | |
| Population group | PWID | MSM/TG* |  | PWID | MSM/TG** |
| Sample size | (n=10) | (n=10) |  | (n=10) | (n=10) |
| Median age, years (IQR) | 39 (32-43) | 26 (21-30) |  | 40 (31-45) | 29 (24-35) |
| Gender, Male | 100% | 90% |  | 90% | 80% |
| Educational level |  |  |  |  |  |
| Below high school | 10% | 10% |  | 0% | 0% |
| High school | 80% | 50% |  | 50% | 30% |
| Undergraduate | 10% | 40% |  | 50% | 70% |
| Employment |  |  |  |  |  |
| Employed | 30% | 60% |  | 30% | 70% |
| Unemployed | 70% | 40% |  | 70% | 30% |
| Marital status |  |  |  |  |  |
| Married/living with a partner | 60% | 10% |  | 40% | 0% |
| Single | 20% | 90% |  | 60% | 70% |
| Divorced or widowed | 20% | 0% |  | 0% | 30% |

*One TG participated in the cognitive interview

**Two TG participated in the in-depth interview
