# Supplementary material for: Usability and acceptability of oral-based HCV self-testing among key populations: a mixed-methods evaluation in Tbilisi, Georgia
Source: BMC Infect Dis. 2022 May 31;22:510. doi: 10.1186/s12879-022-07484-2 (PMC9154030; doi:10.1186/s12879-022-07484-2)
Supplement: Supplementary file 9 — Additional file 9. In-depth post-testing interview topics and quotations. [file 12879_2022_7484_MOESM9_ESM.docx]

**Supplement 6** **Findings from in-depth interviews on PWIDs’ and MSM/TG’s perspectives on HCVST**

**Usability and acceptability of oral-based HCV self-testing among key populations: A mixed-methods evaluation in Tbilisi, Georgia**

Emmanuel Fajardo, Victoria Watson, Moses Kumwenda, Dali Usharidze, Sophiko Gogochashvili, David Kakhaberi, Ana Giguashvili, Cheryl C Johnson, Muhammad S Jamil, Russell Dacombe, Ketevan Stvilia Philippa Easterbrook, Elena Ivanova Reipold.

| **Domain** | **Theme** | **Rationale** | **Example quotation** |
| --- | --- | --- | --- |
| Barriers | Fear | Fear of having the virus may be a potential barrier to access HCV testing services. | “First of all, it is fear, they are afraid to find out that they have C (Hepatitis C).” [113, PWID]. |
|  | Shame | Potential barrier to access HCV testing services. | “I know lot of people who don’t go for testing because they are ashamed if the test results will be positive.” [118, PWID]. |
|  | Social  stigma | Sense of shame associated with HCV infection and was often cited as an example by several MSM participants. Perceived as a risk to the employment of the infected individual or being seen accessing HCV testing facilities by someone they knew and the way this would impact on their social standing. | “You know what can be the reason, if they are employed in governmental structures its possible, they avoid testing because fear to lose the job, there is risk of it.” [008, MSM]. |
|  |  |  | “There are lot of cases when people don’t do tests in laboratory, because they are afraid that somebody can see them in clinics and laboratories, it does not matter if person tests positive or negative and being seen in the clinics just that can be a stigma.” [011, MSM]. |
|  | Denial | Some people may not believe they could contract or be infected with HCV, especially if they were asymptomatic. | “…they have risky behaviour and they know that maybe they are infected, but prefer not to know and be calm, in denial.” [003, MSM]. |
|  | Low  motivation | Men less motivated to access HCV testing and self-care than women. | “I think more women because men usually don’t take care of health. But it depends on a person.” [114, PWID]. |
|  | Treatment  side effects | Raised by few participants as a reason that others might not access testing. These included heart, liver, stomach, and urinary tract problems as well as, psychological problems and death. | “…but also, many rumours or facts I don’t know, exist that say these medicines cause other health problems, like heart attack etc.” [012, MSM]. |
|  |  |  | “…but the fact is a person was alive, started treatment and he died, what was the reason I don’t know.” [113, PWID]. |
|  | Financial | Cost of the test as well as the costs associated with travelling to the clinic to get tested prevents people from accessing testing services. | “I told to this grandmother that as I know it is free. But she said that they asked her grandson 80 GEL for it.” [006, MSM]. |
|  |  |  | “I know he paid 200 GEL before the general analyses and then as I know it is free, but I am not very sure about this.” [113, PWID]. |
| Enablers | Free testing  and availability | Free testing and widespread availability improve the ability of people to get tested. | “Not financial side, because it is free, and it is not a problem.” [116, PWID]. |
| Acceptability | Confidentiality | The removal of the need to physically present at a clinic was seen as an important benefit because it safeguarded individuals from experiencing social stigma. | “...he will do it privately, confidentiality issues are solved with this self-test…” [003, MSM]. |
|  |  |  | “The advantage of self-tests is using it anonymously; lots of community members does not want to be affiliated to community organizations and many people will order it online.” [012, MSM]. |
|  | Ease  of use | Participants found the HCV oral fluid-based self-test easy to use. The short time it took to perform and interpret the test was also seen as an advantage over the current testing system. | “It is easier, and you don’t have to contact blood, there is no need to have a contact with needle. I’m saying this from doctors’ point of view as well as from patient’s point of view. This test is easier.” [004, MSM]. |
|  |  |  | “The process of opening is wonderful. There are little cuts, from where you should tear it. A 2-year-old child could do that. Packing, everything is wonderful.” [004, MSM]. |
|  | Ease  of access | The lack of need to visit a clinic was seen as reducing the barriers associated with accessing HCV testing clinics such as travel costs and social stigma. | “Me and my friend have always been talking to each other, ‘let’s go and do the test, let’s go to Justice house’ But till now, we could not have managed to do so. Now, thanks to your organization, I managed to do the test.” [118, PWID]. |
|  | Ease of sampling | Oral HCV self-test is less invasive and less painful when compared to the available testing approaches in the routine health facilities. | “Yes, blood test is painful this is not painful, this is better.” [116, PWID]. |
|  |  |  | “Many people try to avoid needle. They think that this needle may be used again and again or somehow is contaminated with virus and trying to protect themselves”. [008, MSM]. |
| Concerns | Test  accuracy | Participants were unsure of the accuracy of test and citing a lack of information about the test performance. Users ability to effectively perform a test also contributed towards accuracy concerns towards HCV self-tests. | “When we talk about quick tests and especially about oral tests, you cannot be 100% sure.” [012, MSM]. |
|  |  |  | “I trust blood test more. But it is not absolute as well. The right way is to check you permanently – every three months.” [012, MSM]. |
|  |  |  | “I’m not sure because I have no information. How trustworthy are these tests?” [013, MSM]. |
|  | Cost of the  HCV self-test | Important considering that many Georgians struggle financially with basic needs. Should be in line with other self-tests such as pregnancy tests. Affordable price being 10 Georgian Lari (US$3.50). | “I think up to 5 GEL; somewhere 5-10 GEL, not much more than 5 GEL.” [004, MSM]. |
|  |  |  | “I said it should be somewhere up to 10 GEL. If it will be cheaper it will be better.” [118, MSM]. |
|  | Linkage  to care | Linkage to formal healthcare after a positive test, seemed difficult in the LGBTQ+ group possibly because of the amplified levels of stigma. | “It is better with peer educator, because when I saw that the test result on HIV was positive and the second line became visible, my curator started explaining that HIV is manageable disease.” [011, MSM]. |
| Knowledge | Infection / disease | Although identified as an infectious disease, most participants stated that they knew very little about HCV. | “I don’t know anything; I just know that this is disease and if you don’t treat it your liver will be damaged” [004, MSM]. |
|  |  |  | “Biggest problem is lack of information. If everybody knew that testing is easy for HCV many would do it.” [008, MSM]. |
|  | Transmission | Most participants identified and recognised routes of transmission for HCV. | “It can be transmitted with many ways, with syringe, with tooth, with saliva, with not sterile equipment which are somehow attached with blood or with saliva. I know that it is not transmitted with kissing and caressing” [114, PWID]. |
|  |  |  | “Any circumstances, I mean, everybody should test. If a person is suspicious about past dental procedures or had unprotected sex with strangers or used shared syringe. Any person if they have these risk factors should go and get tested” [005, MSM]. |
|  | Access to  testing | Participants listed the AIDS centre, NGOs, clinics, doctor surgeries, and hospitals as places to access HCV testing services. Participants stated that symptoms are a reason for going, and a lack of symptoms is a reason for not going. Socioeconomic status may determine the ability to seek HCV testing services. | “If people don’t have symptoms, they don’t go, just think, that they will not have it” [002, MSM]. |
|  |  |  | “If we talk about target groups – it should be IDUs, but I think it also should be implemented for general population – as it is done abroad that they make checking every 6 months it will be great here – in Georgia as well” [117, PWID]. |
|  |  |  | “Economic status – I think middle income people have more information and they go more often” [003, MSM]. |
|  | Access to treatment | Most individuals knew that there was treatment for chronic HCV infection. Some participants were specific enough to indicate that HCV treatment in Georgia was provided through the national HCV elimination programme. | “There are some programs, as I know if a person is C positive it become participant of the program and receives the medicine for free, which cures him” [113, PWID]. |
|  |  |  | “I just have information about program of Hepatitis which is in Georgia, and which means that treatment is free; some barriers still are present, but it is free” [012, MSM]. |
|  |  |  | “If you want to know my opinion, we should pray for our former prime minister [..], because the elimination program which we have in Georgia. It is his work. And around me I know lot of people – half Tbilisi were survived” [118, PWID]. |
